# Supplementary material for: Grant writing coaching groups: A randomized trial to discern the influences of coaching duration and engagement of scientific mentors on participants’ proposal submission and success rates
Source: PLoS One. 2026 Nov 10;21(11):e0355883. doi: 10.1371/journal.pone.0355883 (PMC13750578; doi:10.1371/journal.pone.0355883)
Supplement: Supplementary file 1 [file pone.0355883.s001.docx]

| **S1 Table. Awards by Source (and Mechanism for NIH) for Faculty and Postdocs.** | | | | |
| --- | --- | --- | --- | --- |
| **Funding Agency (and Mechanism for NIH) by Analysis Category** | | **Faculty** | **Postdocs** | **All** |
| NIH R01 | | 31 | 0 | 31 |
| NIH/NSF R01-like | |  |  |  |
|  | DP1 | 1 | 0 | 1 |
|  | DP2 | 2 | 0 | 2 |
|  | P20 | 1 | 0 | 1 |
|  | P50 | 0 | 1 | 1 |
|  | R15 | 5 | 0 | 5 |
|  | R16 | 1 | 0 | 1 |
|  | R35 | 6 | 0 | 6 |
|  | R37 | 3 | 0 | 3 |
|  | R56 | 2 | 0 | 2 |
|  | R61 | 1 | 0 | 1 |
|  | U01 | 1 | 0 | 1 |
|  | U19 | 1 | 0 | 1 |
|  | NSF | 7 | 0 | 7 |
|  | *Total R01-like* | *31* | *1* | *32* |
| NIH Other Research | |  |  |  |
|  | R03 | 6 | 0 | 6 |
|  | R21 | 5 | 0 | 5 |
|  | R25 | 2 | 0 | 2 |
|  | R34 | 1 | 0 | 1 |
|  | R43 | 1 | 0 | 1 |
|  | Supplement | 1 | 2 | 3 |
|  | *Total NIH Other Research* | *16* | *2* | *18* |
| NIH K | |  |  |  |
|  | K01 | 6 | 8 | 14 |
|  | K08 | 3 | 1 | 4 |
|  | K12 | 1 | 2 | 3 |
|  | K18 | 1 | 0 | 1 |
|  | K23 | 8 | 3 | 11 |
|  | K76 | 0 | 1 | 1 |
|  | K99 | 0 | 2 | 2 |
|  | KL2 | 2 | 0 | 2 |
|  | *Total NIH K* | *21* | *17* | *38* |
| Non-NIH/NSF Federal | |  |  |  |
|  | ACL (Administration for Community Living) | 1 | 0 | 1 |
|  | DOD (Department of Defense) | 1 | 0 | 1 |
|  | NASA (National Aeronautics and Space Administration) | 1 | 1 | 2 |
|  | PCORI (Patient-Centered Outcomes Research Institute) | 3 | 0 | 3 |
|  | USDA (US Department of Agriculture) | 4 | 0 | 4 |
|  | US Army | 2 | 0 | 2 |
|  | AHRQ (Agency for Health Research and Quality) | 2 | 0 | 2 |
|  | *Total Non-NIH/NSF Federal* | *14* | *1* | *15* |
| National Foundation | |  |  |  |
|  | Alzheimer's Association | 1 | 0 | 1 |
|  | American Academy of Sleep Medicine Foundation | 0 | 1 | 1 |
|  | Helmsley Charitable Trust | 0 | 1 | 1 |
|  | Robert Wood Johnson Foundation | 1 | 0 | 1 |
|  | American Heart Association | 2 | 0 | 2 |
|  | Other National Foundation | 5 | 2 | 7 |
|  | *Total National Foundation* | *9* | *4* | *13* |
| **Total Awards** | | **122** | **25** | **147** |
| NIH = National Institutes of Health, NSF = National Science Foundation | | | | |
