# Supplementary material for: Grant writing coaching groups: A randomized trial to discern the influences of coaching duration and engagement of scientific mentors on participants’ proposal submission and success rates
Source: PLoS One. 2026 Nov 10;21(11):e0355883. doi: 10.1371/journal.pone.0355883 (PMC13750578; doi:10.1371/journal.pone.0355883)
Supplement: Supplementary file 2 [file pone.0355883.s002.docx]

| **S2 Table.** **Descriptive Statistics for Submission Outcomes (Primary and Secondary).** | | | | | | |
| --- | --- | --- | --- | --- | --- | --- |
| **Outcomes** | **Total** | **Regular Dose**  **Unstructured** | **Regular Dose**  **Structured** | **Extended Dose**  **Unstructured** | **Extended Dose**  **Structured** |  |
| **Panel A. All Participants** | (n = 367) | (n=90) | (n = 93) | (n = 92) | (n = 92) |  |
| **Primary** |  |  |  |  |  |  |
| Any submission | 254 (69%) | 59 (66%) | 64 (69%) | 62 (67%) | 69 (75%) |  |
| **Secondary** |  |  |  |  |  |  |
| Any K | 80 (22%) | 15 (17%) | 26 (28%) | 22 (24%) | 17 (18%) |  |
| Any Other NIH Research | 77 (21%) | 20 (22%) | 20 (22%) | 18 (20%) | 19 (21%) |  |
| Any Other Federal Research | 11 (3%) | 5 (6%) | 2 (2%) | 1 (1%) | 3 (3%) |  |
| Any National Foundation | 62 (17%) | 13 (14%) | 17 (18%) | 13 (14%) | 19 (21%) |  |

| **Panel B. Faculty** | (n = 271) | (n = 68) | (n = 71) | (n = 66) | (n = 66) |
| --- | --- | --- | --- | --- | --- |
| **Primary** |  |  |  |  |  |
| Any submission | 198 (73%) | 46 (68) | 51 (72%) | 48 (73%) | 53 (80%) |
| **Secondary** |  |  |  |  |  |
| Any R01 or R01-like | 119 (44%) | 28 (41%) | 27 (38%) | 30 (45%) | 34 (52%) |
| Any R01 | 97 (36%) | 21 (31%) | 22 (31%) | 23 (35%) | 21 (32%) |
| Any R01-like | 52 (19%) | 10 (15%) | 9 (13%) | 11 (17%) | 22 (33%) |
| Any K | 41 (15%) | 7 (10%) | 15 (21%) | 10 (15%) | 9 (14%) |
| Any Other NIH Research | 67 (25%) | 17 (25%) | 17 (24%) | 18 (27%) | 15 (23%) |
| Any Other Federal Research | 10 (4%) | 4 (6%) | 2 (3%) | 1 (2%) | 3 (5%) |
| Any National Foundation | 48 (18%) | 9 (13%) | 15 (21%) | 10 (15%) | 14 (21%) |

| **Panel C. Postdocs** | (n = 96) | (n = 22) | (n = 22) | (n = 26) | (n = 26) | |
| --- | --- | --- | --- | --- | --- | --- |
| **Primary** |  |  |  |  |  | |
| Any submission | 56 (58%) | 13 (59%) | 13 (59%) | 14 (54%) | 16 (62%) | |
| **Secondary** |  |  |  |  |  | |
| Any K | 39 (41%) | 8 (36%) | 11 (50%) | 12 (46%) | 8 (31%) | |
| Any Other NIH Research | 10 (10%) | 3 (14%) | 3 (14%) | 0 (0%) | 4 (15%) | |
| Any Other Federal Research | 1 (1%) | 1 (5%) | 0 (0%) | 0 (0%) | 0 (0%) | |
| Any National Foundation | 14 (15%) | 4 (18%) | 2 (9%) | 3 (12%) | 5 (19%) | |
| Data are presented as numbers with percentages (%) in parentheses. | | | | | |  |
