# Supplementary material for: Grant writing coaching groups: A randomized trial to discern the influences of coaching duration and engagement of scientific mentors on participants’ proposal submission and success rates
Source: PLoS One. 2026 Nov 10;21(11):e0355883. doi: 10.1371/journal.pone.0355883 (PMC13750578; doi:10.1371/journal.pone.0355883)
Supplement: Supplementary file 3 [file pone.0355883.s003.docx]

| **S3 Table. Descriptive Statistics for Award Outcomes (Primary and Secondary).** | | | | | |  |
| --- | --- | --- | --- | --- | --- | --- |
| **Outcomes** | **Total** | **Regular Dose**  **Unstructured** | **Regular Dose**  **Structured** | **Extended Dose**  **Unstructured** | **Extended Dose**  **Structured** | |
| **Panel A. All Participants** | (n = 367) | (n = 90) | (n = 93) | (n = 92) | (n = 92) | |
| **Primary** |  |  |  |  |  | |
| Any award | 116 (32%) | 22 (24%) | 29 (31%) | 25 (27%) | 40 (43%) | |
| **Secondary** |  |  |  |  |  | |
| Any K | 38 (10%) | 5 (6%) | 14 (15%) | 8 (9%) | 11 (12%) | |
| Any Other NIH Research | 18 (5%) | 3 (3%) | 4 (4%) | 5 (5%) | 6 (7%) | |
| Any Other Federal Research | 11 (3%) | 5 (6%) | 2 (2%) | 1 (1%) | 3 (3%) | |
| Any National Foundation | 13 (4%) | 0 (0%) | 3 (3%) | 5 (5%) | 5 (5%) | |

| **Panel B. Faculty** | (n = 271) | (n = 68) | (n = 71) | (n = 66) | (n = 66) |
| --- | --- | --- | --- | --- | --- |
| **Primary** |  |  |  |  |  |
| Any award | 95 (35%) | 17 (25%) | 24 (34%) | 20 (30%) | 34 (52%) |
| **Secondary** |  |  |  |  |  |
| Any R01 or R01-like | 55 (20%) | 10 (15%) | 11 (15%) | 13 (20%) | 21 (32%) |
| Any R01 | 28 (10%) | 6 (9%) | 7 (10%) | 8 (12%) | 7 (11%) |
| Any R01-like | 28 (10%) | 4 (6%) | 4 (6%) | 6 (9%) | 14 (21%) |
| Any K | 21 (8%) | 1 (1%) | 10 (14%) | 4 (6%) | 6 (9%) |
| Any Other NIH Research | 16 (6%) | 3 (4%) | 3 (4%) | 5 (8%) | 5 (8%) |
| Any Other Federal Research | 10 (4%) | 4 (6%) | 2 (3%) | 1 (2%) | 3 (5%) |
| Any National Foundation | 9 (3%) | 0 (0%) | 2 (3%) | 3 (5%) | 4 (6%) |

| **Panel C. Postdocs** | (n = 96) | (n = 22) | (n = 22) | (n = 26) | (n = 26) | |
| --- | --- | --- | --- | --- | --- | --- |
| **Primary** |  |  |  |  |  | |
| Any award | 21 (22%) | 5 (23%) | 5 (23%) | 5 (19%) | 6 (23%) | |
| **Secondary** |  |  |  |  |  | |
| Any K | 17 (18%) | 4 (18%) | 4 (18%) | 4 (15%) | 5 (19%) | |
| Any Other NIH Research | 2 (2%) | 0 (0%) | 1 (5%) | 0 (0%) | 1 (4%) | |
| Any Other Federal Research | 1 (1%) | 1 (5%) | 0 (0%) | 0 (0%) | 0 (0%) | |
| Any National Foundation | 4 (4%) | 0 (0%) | 1 (5%) | 2 (8%) | 1 (4%) | |
| Data are presented as numbers with percentages (%) in parentheses. | | | | | |  |
