# Supplementary material for: Grant writing coaching groups: A randomized trial to discern the influences of coaching duration and engagement of scientific mentors on participants’ proposal submission and success rates
Source: PLoS One. 2026 Nov 10;21(11):e0355883. doi: 10.1371/journal.pone.0355883 (PMC13750578; doi:10.1371/journal.pone.0355883)
Supplement: Supplementary file 4 [file pone.0355883.s004.docx]

| **S4 Table.** **Descriptive Statistics for Success Outcomes (Primary and Secondary).** | | | | | | |
| --- | --- | --- | --- | --- | --- | --- |
| **Outcomes** | **Total** | **Regular Dose**  **Unstructured** | **Regular Dose**  **Structured** | **Extended Dose**  **Unstructured** | **Extended Dose**  **Structured** |  |
| **Panel A. All Participants** |  |  |  |  |  |  |
| **Primary** |  |  |  |  |  |  |
| Any success | 116/254 (46%) | 22/59 (37%) | 29/64 (45%) | 25/62 (40%) | 40/69 (58%) |  |
| **Secondary** |  |  |  |  |  |  |
| Any K | 38/80 (48%) | 5/15 (33%) | 14/26 (54%) | 8/22 (36%) | 11/17 (65%) |  |
| Any Other NIH Research | 18/77 (23%) | 3/20 (15%) | 4/20 (20%) | 5/18 (28%) | 6/19 (32%) |  |
| Any Other Federal Research | 11/11 (100%) | 5/5 (100%) | 2/2 (100%) | 1/1 (100%) | 3/3 (100%) |  |
| Any National Foundation | 13/62 (21%) | 0/13 (0%) | 3/17 (18%) | 5/13 (38%) | 5/19 (26%) |  |

| **Panel B. Faculty** |  |  |  |  |  |
| --- | --- | --- | --- | --- | --- |
| **Primary** |  |  |  |  |  |
| Any success | 95/198 (48%) | 17/46 (37%) | 24/51 (47%) | 20/48 (42%) | 34/53 (64%) |
| **Secondary** |  |  |  |  |  |
| Any R01 or R01-like | 55/119 (46%) | 10/28 (36%) | 11/27 (41%) | 13/30 (43%) | 21/34 (62%) |
| Any R01 | 28/87 (32%) | 6/21 (29%) | 7/22 (32%) | 8/23 (35%) | 7/21 (33%) |
| Any R01-like | 28/52 (54%) | 4/10 (40%) | 4/9 (44%) | 6/11 (55%) | 14/22 (64%) |
| Any K | 21/41 (51%) | 1/7 (14%) | 10/15 (67%) | 4/10 (40%) | 6/9 (67%) |
| Any Other NIH Research | 16/67 (24%) | 3/17 (18%) | 3/17 (18%) | 5/18 (28%) | 5/15 (33%) |
| Any Other Federal Research | 10/10 (100%) | 4/4 (100%) | 2/2 (100%) | 1/1 (100%) | 3/3 (100%) |
| Any National Foundation | 9/48 (19%) | 0/9 (0%) | 2/15 (13%) | 3/10 (30%) | 4/14 (29%) |

| **Panel C. Postdocs** |  |  |  |  |  |
| --- | --- | --- | --- | --- | --- |
| **Primary** |  |  |  |  |  |
| Any success | 21/56 (38%) | 5/13 (38%) | 5/13 (38%) | 5/14 (36%) | 6/16 (38%) |
| **Secondary** |  |  |  |  |  |
| Any K | 17/39 (44%) | 4/8 (50%) | 4/11 (36%) | 4/12 (33%) | 5/8 (63%) |
| Any Other NIH Research | 2/10 (20%) | 0/3 (0%) | 1/3 (33%) | 0/0 (-) | 1/4 (25%) |
| Any Other Federal Research | 1/1 (100%) | 1/1 (100%) | 0/0 (-) | 0/0 (-) | 0/0 (-) |
| Any National Foundation | 4/14 (29%) | 0/4 (0%) | 1/2 (50%) | 2/3 (67%) | 1/5 (20%) |
| Data are presented as numbers with percentages (%) in parentheses. Success = any national-level award among those with any national-level submission of corresponding grant type. | | | | | |
