# Supplementary material for: Grant writing coaching groups: A randomized trial to discern the influences of coaching duration and engagement of scientific mentors on participants’ proposal submission and success rates
Source: PLoS One. 2026 Nov 10;21(11):e0355883. doi: 10.1371/journal.pone.0355883 (PMC13750578; doi:10.1371/journal.pone.0355883)
Supplement: Supplementary file 5 [file pone.0355883.s005.docx]

| **S5 Table. Extended Dose Responders to 24-Month Coaching Survey.** | | | | | | |
| --- | --- | --- | --- | --- | --- | --- |
|  | **Faculty Responders in Extended Dose** | | | **Postdoc Responders in Extended Dose** | | |
|  | Total  (n=66) | Structured  (n=36) | Unstructured  (n=30) | Total  (n=19) | Structured  (n=10) | Unstructured  (n=9) |
| **Summary of Coach Support Among Responders** |  |  |  |  |  |  |
| Coach provided at least 1 type of support | 52 (79%) | 31 (86%) | 21 (70%) | 12 (63%) | 5 (50%) | 7 (78%) |
| No coach support | 14 (21%) | 5 (14%) | 9 (30%) | 7 (37%) | 5 (50%) | 2 (22%) |
| **Type of Coach Support (choose all that apply)** |  |  |  |  |  |  |
| 1. Gave me feedback on drafts to support initial (first) submission of my proposal | 33 (50%) | 19 (53%) | 13 (43%) | 8 (42%) | 3 (30%) | 5 (56%) |
| 2. Helped me interpret the NIH Summary Statement reviews that I received for my proposal | 22 (33%) | 13 (36%) | 9 (30%) | 4 (21%) | 2 (20%) | 2 (22%) |
| 3. Gave me feedback on drafts to support resubmission of my proposal | 11 (17%) | 8 (22%) | 3 (10%) | 2 (11%) | 2 (20%) | 0 |
| 4. Helped me identify scientific issues that needed additional input from an expert | 18 (27%) | 12 (33%) | 6 (20%) | 2 (11%) | 1 (10%) | 1 (11%) |
| 5. Helped with other professional development needs | 15 (23%) | 11 (31%) | 4 (13%) | 2 (11%) | 2 (20%) | 0 |
