# Supplementary material for: Grant writing coaching groups: A randomized trial to discern the influences of coaching duration and engagement of scientific mentors on participants’ proposal submission and success rates
Source: PLoS One. 2026 Nov 10;21(11):e0355883. doi: 10.1371/journal.pone.0355883 (PMC13750578; doi:10.1371/journal.pone.0355883)
Supplement: Supplementary file 6 [file pone.0355883.s006.docx]

| **S6 Table.** **All Participants: Multivariable Logistic Regression Models.** | | | | | | | | |
| --- | --- | --- | --- | --- | --- | --- | --- | --- |
|  | Any submission | | | | Any K submission | | | |
| **Panel A. Submissions** | OR | 95% CI | | *P*-value^a^ | OR | 95% CI | | *P*-value |
| **Structured vs Unstructured** | 1.04 | 0.64 | 1.70 | 0.876 | 0.85 | 0.47 | 1.53 | 0.586 |
| **Extended vs Regular Dose** | 1.08 | 0.67 | 1.76 | 0.752 | 0.88 | 0.50 | 1.55 | 0.659 |
| Mid/late vs early career | 1.57 | 0.53 | 4.60 | 0.415 | 0.18 | 0.02 | 1.48 | 0.111 |
| Postdoc vs early career | 0.74 | 0.41 | 1.34 | 0.318 | 2.86 | 1.49 | 5.50 | **0.002** |
| URM vs non-URM | 0.60 | 0.37 | 0.99 | **0.045** | 0.88 | 0.50 | 1.56 | 0.661 |
| Female vs Male | 1.18 | 0.69 | 2.00 | 0.546 | 0.87 | 0.46 | 1.65 | 0.675 |
| Other vs Male | NE | NE | NE | 0.988 | 3.83 | 0.54 | 26.93 | 0.177 |
| Clinical vs Lab | 1.13 | 0.58 | 2.22 | 0.723 | 4.43 | 2.09 | 9.38 | **<0.001** |
| Epi/DS vs Lab | 0.76 | 0.33 | 1.75 | 0.520 | 1.69 | 0.61 | 4.70 | 0.314 |
| Soc/Beh vs Lab | 0.75 | 0.41 | 1.38 | 0.358 | 1.28 | 0.61 | 2.68 | 0.521 |
| Any prior training submissions | 1.81 | 1.10 | 2.98 | **0.020** | 1.34 | 0.73 | 2.43 | 0.345 |
| Any prior research submissions | 1.57 | 0.89 | 2.77 | 0.122 | 1.41 | 0.72 | 2.77 | 0.323 |
| Age | 1.01 | 0.97 | 1.05 | 0.619 | 0.97 | 0.92 | 1.02 | 0.226 |
| Number prior publications | 1.01 | 0.99 | 1.03 | 0.437 | 0.96 | 0.93 | 0.99 | **0.005** |

|  | Any award | | | | Any K award | | | |
| --- | --- | --- | --- | --- | --- | --- | --- | --- |
| **Panel B. Awards** | OR | 95% CI | | *P*-value | OR | 95% CI | | *P*-value |
| **Structured vs Unstructured** | 1.46 | 0.89 | 2.38 | 0.133 | 1.69 | 0.78 | 3.68 | 0.185 |
| **Extended vs Regular Dose** | 1.21 | 0.75 | 1.96 | 0.436 | 1.01 | 0.48 | 2.11 | 0.989 |
| Mid/late vs early career | 1.72 | 0.72 | 4.10 | 0.219 | 0.57 | 0.07 | 4.83 | 0.608 |
| Postdoc vs early career | 0.63 | 0.33 | 1.20 | 0.161 | 1.92 | 0.83 | 4.47 | 0.130 |
| URM vs non-URM | 0.83 | 0.51 | 1.36 | 0.453 | 1.11 | 0.53 | 2.32 | 0.780 |
| Female vs Male | 1.16 | 0.69 | 1.97 | 0.574 | 1.19 | 0.49 | 2.89 | 0.706 |
| Other vs Male | 1.22 | 0.17 | 8.59 | 0.842 | 7.82 | 1.03 | 59.58 | **0.047** |
| Clinical vs Lab | 0.76 | 0.41 | 1.43 | 0.397 | 2.42 | 0.92 | 6.37 | 0.073 |
| Epi/DS vs Lab | 0.45 | 0.18 | 1.14 | 0.093 | 0.99 | 0.19 | 5.23 | 0.993 |
| Soc/Beh vs Lab | 0.68 | 0.38 | 1.24 | 0.212 | 1.56 | 0.60 | 4.06 | 0.365 |
| Any prior training submissions | 1.46 | 0.87 | 2.43 | 0.153 | 0.66 | 0.31 | 1.41 | 0.285 |
| Any prior research submissions | 1.39 | 0.75 | 2.60 | 0.297 | 1.53 | 0.63 | 3.73 | 0.346 |
| Age | 0.96 | 0.92 | 1.00 | 0.058 | 0.97 | 0.91 | 1.03 | 0.362 |
| Number prior publications | 1.02 | 1.00 | 1.03 | 0.056 | 0.97 | 0.93 | 1.00 | 0.083 |

|  | Any success | | | | Any K success | | | |
| --- | --- | --- | --- | --- | --- | --- | --- | --- |
| **Panel C. Success** | OR | 95% CI | | *P*-value | OR | 95% CI | | *P*-value |
| **Structured vs Unstructured** | 1.57 | 0.91 | 2.71 | 0.104 | 3.29 | 1.00 | 10.81 | 0.050 |
| **Extended vs Regular Dose** | 1.26 | 0.74 | 2.15 | 0.392 | 1.60 | 0.57 | 4.49 | 0.373 |
| Mid/late vs early career | 1.55 | 0.60 | 3.98 | 0.362 | NE | NE | NE | 0.985 |
| Postdoc vs early career | 0.75 | 0.37 | 1.54 | 0.438 | 0.93 | 0.29 | 2.99 | 0.907 |
| URM vs non-URM | 1.13 | 0.65 | 1.97 | 0.666 | 1.16 | 0.39 | 3.43 | 0.789 |
| Female vs Male | 1.09 | 0.61 | 1.95 | 0.776 | 3.39 | 0.95 | 12.06 | 0.059 |
| Other vs Male | 0.60 | 0.09 | 4.22 | 0.606 | NE | NE | NE | 0.979 |
| Clinical vs Lab | 0.66 | 0.33 | 1.33 | 0.242 | 0.40 | 0.10 | 1.65 | 0.204 |
| Epi/DS vs Lab | 0.44 | 0.16 | 1.21 | 0.112 | 0.33 | 0.04 | 2.60 | 0.291 |
| Soc/Beh vs Lab | 0.75 | 0.38 | 1.48 | 0.406 | 1.02 | 0.23 | 4.46 | 0.978 |
| Any prior training submissions | 1.20 | 0.67 | 2.14 | 0.533 | 0.40 | 0.13 | 1.19 | 0.100 |
| Any prior research submissions | 1.29 | 0.65 | 2.55 | 0.473 | 2.07 | 0.58 | 7.31 | 0.261 |
| Age | 0.95 | 0.90 | 0.99 | **0.025** | 1.02 | 0.93 | 1.12 | 0.631 |
| Number prior publications | 1.02 | 1.00 | 1.04 | 0.063 | 1.01 | 0.95 | 1.07 | 0.842 |
| Any success = any national-level award among those with any national-level submission of corresponding grant type. OR = adjusted odds ratio. NE = not estimable for OR (95% CI) due to small sample size. URM = underrepresented in medicine. Epi/DS = epidemiology/data science research focus. Soc/Beh = social/behavioral science research focus.  ^a^*P*-value is from logistic regression’s two-sided Wald test. | | | | | | | | |
