# Supplementary material for: Grant writing coaching groups: A randomized trial to discern the influences of coaching duration and engagement of scientific mentors on participants’ proposal submission and success rates
Source: PLoS One. 2026 Nov 10;21(11):e0355883. doi: 10.1371/journal.pone.0355883 (PMC13750578; doi:10.1371/journal.pone.0355883)
Supplement: Supplementary file 7 [file pone.0355883.s007.docx]

**S7 Table. Faculty: Multivariable Logistic Regression Models.**

|  | Any submission | | | | Any R01 or R01-like submission | | | | Any K submission | | | |
| --- | --- | --- | --- | --- | --- | --- | --- | --- | --- | --- | --- | --- |
| **Panel A. Submissions** | OR | 95% CI | | *P*-value^a^ | OR | 95% CI | | *P* | OR | 95% CI | | *P* |
| Structured vs Uns | 1.15 | 0.64 | 2.06 | 0.642 | 0.89 | 0.51 | 1.55 | 0.679 | 1.23 | 0.57 | 2.67 | 0.602 |
| Extended vs Reg | 1.18 | 0.65 | 2.14 | 0.583 | 1.03 | 0.59 | 1.78 | 0.927 | 1.05 | 0.49 | 2.26 | 0.892 |
| Mid/late vs Early | 1.78 | 0.59 | 5.34 | 0.305 | 2.05 | 0.81 | 5.18 | 0.130 | 0.25 | 0.03 | 2.11 | 0.202 |
| URM vs Non-URM | 0.56 | 0.31 | 1.01 | 0.054 | 0.80 | 0.46 | 1.39 | 0.424 | 0.88 | 0.41 | 1.91 | 0.746 |
| Female vs Male | 1.31 | 0.71 | 2.41 | 0.387 | 1.24 | 0.70 | 2.20 | 0.463 | 0.90 | 0.41 | 2.00 | 0.797 |
| Other vs Male | NE | NE | NE | 0.984 | 5.40 | 0.43 | 67.35 | 0.190 | 2.20 | 0.19 | 25.74 | 0.529 |
| Clinical vs Lab | 1.01 | 0.44 | 2.29 | 0.986 | 0.39 | 0.19 | 0.82 | **0.013** | 6.92 | 2.43 | 19.76 | **<0.001** |
| Epi/DS vs Lab | 0.70 | 0.25 | 1.98 | 0.505 | 0.40 | 0.15 | 1.06 | 0.066 | 3.50 | 0.84 | 14.52 | 0.085 |
| Soc/Beh vs Lab | 0.69 | 0.34 | 1.41 | 0.307 | 0.43 | 0.22 | 0.84 | **0.013** | 2.41 | 0.83 | 6.96 | 0.105 |
| Prior train subs | 1.89 | 1.03 | 3.46 | **0.041** | 2.33 | 1.29 | 4.22 | **0.005** | 0.72 | 0.33 | 1.59 | 0.418 |
| Prior res subs | 1.22 | 0.58 | 2.58 | 0.600 | 1.02 | 0.48 | 2.14 | 0.962 | 1.11 | 0.42 | 2.92 | 0.838 |
| Age | 0.99 | 0.95 | 1.04 | 0.804 | 0.97 | 0.93 | 1.02 | 0.299 | 0.92 | 0.86 | 0.99 | **0.018** |
| Number prior pubs | 1.01 | 0.99 | 1.03 | 0.414 | 1.03 | 1.01 | 1.05 | **0.001** | 0.96 | 0.92 | 0.99 | **0.010** |

|  | Any award | | | | Any R01 or R01-like award | | | | Any K award | | | |
| --- | --- | --- | --- | --- | --- | --- | --- | --- | --- | --- | --- | --- |
| **Panel B.**  **Awards** | OR | 95% CI | | *P* | OR | 95% CI | | *P* | OR | 95% CI | | *P* |
| Structured vs Uns | 1.87 | 1.07 | 3.27 | **0.029** | 1.47 | 0.73 | 2.98 | 0.285 | 3.32 | 1.07 | 10.29 | **0.038** |
| Extended vs Reg | 1.41 | 0.81 | 2.46 | 0.221 | 1.52 | 0.76 | 3.03 | 0.239 | 1.26 | 0.46 | 3.47 | 0.652 |
| Mid/late vs Early | 1.88 | 0.76 | 4.65 | 0.171 | 1.56 | 0.52 | 4.66 | 0.427 | 0.72 | 0.08 | 6.62 | 0.768 |
| URM vs Non-URM | 0.64 | 0.36 | 1.13 | 0.125 | 0.58 | 0.28 | 1.18 | 0.133 | 1.11 | 0.40 | 3.07 | 0.843 |
| Female vs Male | 1.17 | 0.66 | 2.09 | 0.593 | 1.42 | 0.68 | 2.95 | 0.348 | 1.10 | 0.37 | 3.31 | 0.864 |
| Other vs Male | 0.42 | 0.04 | 4.47 | 0.471 | NE | NE | NE | 0.985 | 4.13 | 0.30 | 57.45 | 0.291 |
| Clinical vs Lab | 0.82 | 0.40 | 1.67 | 0.579 | 0.36 | 0.14 | 0.93 | **0.035** | 6.79 | 1.59 | 28.99 | **0.010** |
| Epi/DS vs Lab | 0.50 | 0.18 | 1.40 | 0.185 | 0.61 | 0.19 | 1.95 | 0.400 | 2.34 | 0.20 | 27.05 | 0.495 |
| Soc/Beh vs Lab | 0.68 | 0.34 | 1.34 | 0.264 | 0.51 | 0.21 | 1.23 | 0.134 | 3.01 | 0.68 | 13.42 | 0.148 |
| Prior train subs | 1.45 | 0.80 | 2.62 | 0.226 | 3.43 | 1.44 | 8.15 | **0.005** | 0.52 | 0.19 | 1.47 | 0.219 |
| Prior res subs | 0.80 | 0.38 | 1.69 | 0.564 | 0.85 | 0.33 | 2.23 | 0.744 | 0.94 | 0.25 | 3.49 | 0.924 |
| Age | 0.95 | 0.90 | 1.00 | 0.064 | 0.95 | 0.89 | 1.02 | 0.161 | 0.96 | 0.88 | 1.04 | 0.284 |
| Number prior pubs | 1.02 | 1.00 | 1.03 | 0.075 | 1.04 | 1.02 | 1.06 | **<0.001** | 0.95 | 0.90 | 1.00 | **0.035** |

|  | Any success | | | | Any R01 or R01-like success | | | | Any K success | | | |
| --- | --- | --- | --- | --- | --- | --- | --- | --- | --- | --- | --- | --- |
| **Panel C. Success** | OR | 95% CI | | *P* | OR | 95% CI | | *P* | OR | 95% CI | | *P* |
| Structured vs Uns | 1.95 | 1.04 | 3.65 | **0.037** | 1.90 | 0.79 | 4.58 | 0.153 | 9.58 | 1.19 | 76.92 | **0.034** |
| Extended vs Reg | 1.46 | 0.80 | 2.67 | 0.221 | 1.92 | 0.84 | 4.40 | 0.124 | 1.91 | 0.35 | 10.48 | 0.459 |
| Mid/late vs Early | 1.66 | 0.63 | 4.40 | 0.305 | 1.31 | 0.35 | 4.89 | 0.689 | NE | NE | NE | 0.985 |
| URM vs Non-URM | 0.82 | 0.43 | 1.54 | 0.532 | 0.55 | 0.22 | 1.37 | 0.198 | 1.59 | 0.23 | 10.99 | 0.641 |
| Female vs Male | 1.03 | 0.54 | 1.97 | 0.925 | 1.11 | 0.43 | 2.87 | 0.825 | 2.78 | 0.41 | 18.69 | 0.293 |
| Other vs Male | 0.23 | 0.02 | 2.45 | 0.221 | NE | NE | NE | 0.983 | NE | NE | NE | 0.987 |
| Clinical vs Lab | 0.77 | 0.35 | 1.70 | 0.518 | 0.41 | 0.12 | 1.39 | 0.153 | 1.53 | 0.18 | 13.39 | 0.700 |
| Epi/DS vs Lab | 0.51 | 0.16 | 1.59 | 0.246 | 0.78 | 0.16 | 3.88 | 0.766 | 0.57 | 0.02 | 18.39 | 0.748 |
| Soc/Beh vs Lab | 0.76 | 0.35 | 1.64 | 0.478 | 0.72 | 0.24 | 2.15 | 0.553 | 0.85 | 0.08 | 9.18 | 0.895 |
| Prior train subs | 1.19 | 0.61 | 2.32 | 0.604 | 3.31 | 1.14 | 9.64 | **0.028** | 1.26 | 0.17 | 9.13 | 0.821 |
| Prior res subs | 0.76 | 0.32 | 1.78 | 0.522 | 1.00 | 0.31 | 3.24 | 0.994 | 0.68 | 0.05 | 9.71 | 0.778 |
| Age | 0.95 | 0.89 | 1.00 | 0.061 | 0.95 | 0.87 | 1.03 | 0.212 | 1.29 | 1.01 | 1.64 | **0.041** |
| Number Prior Pubs | 1.02 | 1.00 | 1.04 | 0.114 | 1.03 | 1.00 | 1.06 | **0.037** | 0.93 | 0.84 | 1.04 | 0.206 |
| Any success = any national-level award among those with any national-level submission of corresponding grant type. OR = adjusted odds ratio. NE = not estimable for OR (95% CI) due to small sample size. Uns = unstructured. Reg = regular. Early = early career. URM = underrepresented in medicine. Epi/DS = epidemiology/data science research focus. Soc/Beh = social/behavioral science research focus. Train = training. Res = research. Subs = submissions. Pubs = publications.  ^a^*P*-value is from logistic regression’s two-sided Wald test. | | | | | | | | | | | | |
