# Supplementary material for: Grant writing coaching groups: A randomized trial to discern the influences of coaching duration and engagement of scientific mentors on participants’ proposal submission and success rates
Source: PLoS One. 2026 Nov 10;21(11):e0355883. doi: 10.1371/journal.pone.0355883 (PMC13750578; doi:10.1371/journal.pone.0355883)
Supplement: Supplementary file 8 [file pone.0355883.s008.docx]

| **S8 Table.** **Postdocs: Multivariable Logistic Regression Models.** | | | | | | | | |
| --- | --- | --- | --- | --- | --- | --- | --- | --- |
|  | Any submission | | | | Any K submission | | | |
| ***Panel A. Subs*** | OR | 95% CI | | p | OR | 95% CI | | p |
| Structured vs Unstructured | 0.87 | 0.34 | 2.22 | 0.766 | 0.49 | 0.18 | 1.32 | 0.157 |
| Extended vs Regular Dose | 0.90 | 0.37 | 2.18 | 0.811 | 0.79 | 0.31 | 2.02 | 0.626 |
| URM vs Non-URM | 0.57 | 0.22 | 1.49 | 0.252 | 0.59 | 0.22 | 1.61 | 0.302 |
| Female vs Male | 0.99 | 0.31 | 3.14 | 0.989 | 1.12 | 0.34 | 3.74 | 0.855 |
| Other vs Male | NE | NE | NE | 0.985 | NE | NE | NE | 0.984 |
| Clinical vs Lab | 1.33 | 0.38 | 4.69 | 0.657 | 2.00 | 0.55 | 7.22 | 0.290 |
| Epi/DS vs Lab | 0.87 | 0.21 | 3.65 | 0.843 | 0.70 | 0.15 | 3.30 | 0.656 |
| Soc/Beh vs Lab | 0.88 | 0.28 | 2.78 | 0.822 | 0.56 | 0.16 | 1.96 | 0.366 |
| Any prior training submissions | 1.46 | 0.58 | 3.70 | 0.421 | 2.92 | 1.09 | 7.83 | **0.034** |
| Any prior research submissions | 2.54 | 0.94 | 6.81 | 0.065 | 2.31 | 0.82 | 6.51 | 0.115 |
| Age | 1.06 | 0.98 | 1.14 | 0.158 | 1.05 | 0.96 | 1.14 | 0.286 |
| Number prior publications | 1.00 | 0.94 | 1.06 | 0.862 | 0.96 | 0.90 | 1.02 | 0.207 |

|  | Any award | | | | Any K award | | | |
| --- | --- | --- | --- | --- | --- | --- | --- | --- |
| ***Panel B. Awards*** | OR | 95% CI | | p | OR | 95% CI | | p |
| Structured vs Unstructured | 0.65 | 0.21 | 2.02 | 0.457 | 0.79 | 0.24 | 2.59 | 0.697 |
| Extended vs Regular Dose | 0.74 | 0.25 | 2.21 | 0.584 | 0.76 | 0.24 | 2.37 | 0.638 |
| URM vs non-URM | 1.21 | 0.38 | 3.90 | 0.745 | 0.87 | 0.26 | 2.93 | 0.819 |
| Female vs Male | 1.74 | 0.35 | 8.69 | 0.497 | 2.64 | 0.46 | 15.18 | 0.278 |
| Other vs Male | NE | NE | NE | 0.984 | NE | NE | NE | 0.982 |
| Clinical vs Lab | 0.39 | 0.08 | 1.80 | 0.225 | 0.45 | 0.09 | 2.36 | 0.348 |
| Epi/DS vs Lab | 0.21 | 0.02 | 2.32 | 0.202 | 0.34 | 0.03 | 3.64 | 0.372 |
| Soc/Beh vs Lab | 0.43 | 0.10 | 1.79 | 0.246 | 0.60 | 0.14 | 2.55 | 0.486 |
| Any prior training submissions | 1.30 | 0.41 | 4.09 | 0.652 | 0.86 | 0.26 | 2.83 | 0.806 |
| Any prior research submissions | 5.23 | 1.49 | 18.33 | **0.010** | 3.02 | 0.83 | 11.00 | 0.094 |
| Age | 0.99 | 0.90 | 1.10 | 0.906 | 1.01 | 0.92 | 1.13 | 0.785 |
| Number prior publications | 1.01 | 0.94 | 1.09 | 0.738 | 1.02 | 0.94 | 1.10 | 0.681 |

|  | Any success | | | | Any K success | | | |
| --- | --- | --- | --- | --- | --- | --- | --- | --- |
| ***Panel C. Success*** | OR | 95% CI | | p | OR | 95% CI | | p |
| Structured vs Unstructured | 1.07 | 0.29 | 4.00 | 0.919 | 2.62 | 0.31 | 22.24 | 0.377 |
| Extended vs Regular Dose | 0.71 | 0.19 | 2.71 | 0.617 | 0.77 | 0.12 | 4.94 | 0.785 |
| URM vs Non-URM | 2.14 | 0.47 | 9.82 | 0.328 | 0.90 | 0.12 | 6.86 | 0.922 |
| Female vs Male | 1.96 | 0.31 | 12.63 | 0.477 | 12.78 | 0.72 | 227.29 | 0.083 |
| Other vs Male | NE | NE | NE | 0.985 | NE | NE | NE | 0.977 |
| Clinical vs Lab | 0.30 | 0.05 | 1.80 | 0.188 | 0.07 | 0.00 | 1.10 | 0.058 |
| Epi/DS vs Lab | 0.14 | 0.01 | 2.45 | 0.180 | 0.07 | 0.00 | 2.47 | 0.141 |
| Soc/Beh vs Lab | 0.53 | 0.09 | 3.07 | 0.480 | 1.82 | 0.12 | 26.65 | 0.662 |
| Any prior training submissions | 1.16 | 0.30 | 4.46 | 0.829 | 0.14 | 0.02 | 1.11 | 0.063 |
| Any prior research submissions | 3.53 | 0.87 | 14.23 | 0.077 | 2.00 | 0.24 | 16.50 | 0.519 |
| Age | 0.95 | 0.84 | 1.08 | 0.441 | 0.97 | 0.83 | 1.12 | 0.650 |
| Number prior publications | 1.04 | 0.96 | 1.14 | 0.350 | 1.13 | 0.94 | 1.36 | 0.199 |
| Any success = any award among those with any submission of the corresponding award type. URM = underrepresented in medicine. NE = not estimable for odds ratio (95%) due to small sample size. Epi/DS = epidemiology/data science research focus. Soc/Beh = social/behavioral science research focus.  ^a^*P*-value is from two-sided Wald test from logistic regression. | | | | | | | | |
