# Supplementary material for: Grant writing coaching groups: A randomized trial to discern the influences of coaching duration and engagement of scientific mentors on participants’ proposal submission and success rates
Source: PLoS One. 2026 Nov 10;21(11):e0355883. doi: 10.1371/journal.pone.0355883 (PMC13750578; doi:10.1371/journal.pone.0355883)
Supplement: Supplementary file 9 [file pone.0355883.s009.docx]

| **S9 Table. Simple Effect for Explaining Significant Interactions.** | | | | | | |
| --- | --- | --- | --- | --- | --- | --- |
| Outcome |  |  | Moderator | | Experimental Simple Effects  by Moderator Values | |
|  | Experimental Factor | Inter  *P*-value | Name | Value | OR (95% CI) | *P*-value |
| **Panel A. All Participants** |  |  |  |  |  |  |
| **Submissions** |  |  |  |  |  |  |
| Any K | Structured vs Unstructured | **.043** | Duration | Extended  Regular | 0.46 (0.20,1.07)  1.50 (0.67,3.37) | .072  .329 |
|  | Extended vs  Regular Dose |  | SA engagement Mode | Structured  Unstructured | 0.50 (0.22,1.11)  1.61 (0.71,3.63) | .086  .256 |
| Any | Structured vs unstructured | **.031** | Previous training submissions | Yes  No | 0.64 (0.33,1.25)  1.89 (0.90,3.95) | .192  .093 |
| Any K | Structured vs Unstructured | **.044** | Previous training submissions | Yes  No | 0.52 (0.25,1.11)  1.68 (0.70,4.06) | .091  .250 |
| Any K | Structured vs unstructured | **.016** | Previous research submissions | Yes  No | 0.53 (0.26,1.07)  2.32 (0.84,6.38) | .078  .103 |
| **Awards** |  |  |  |  |  |  |
| Any K | Structured vs Unstructured | **.039** | Previous research submissions | Yes  No | 1.02 (0.43,2.45)  7.08 (1.35,37.20) | .962  **.021** |
| **Success** | | | | | | |
| Any | Structured vs Unstructured | **.020** | URM status | URM  Non-URM | 3.43 (1.44,8.18)  .93 (0.46,1.88) | **.005**  .846 |
| Any | Extended vs  Regular Dose | **.045** | Previous research submissions | Yes  No | 1.68 (0.92,3.07)  0.46 (0.15,1.42) | .093  .178 |
| Any K | Structured vs Unstructured | **.045** | Number of prior publications | Q1 (pubs=6)  Q2 (pubs=11.5)  Q3 (pubs=16) | 1.32 (0.31,5.69)  2.90 (0.83,10.06)  **5.50 (1.36,22.25)** | NA |

| **Panel B. Faculty** |  |  |  |  |  |  |
| --- | --- | --- | --- | --- | --- | --- |
| **Submissions (no significant interactions)** | |  |  |  |  |  |
| **Awards** |  |  |  |  |  |  |
| Any R01 or R01-Like | Structured vs unstructured | **.030** | URM status | URM  Non-URM | 4.32 (1.24, 14.98)  0.81 (0.33, 1.96) | **.021**  .636 |
| Any R01 or R01-Like | Extended vs  Regular Dose | **.029** | Previous research submissions | Yes  No | 2.13 (0.99, 4.60)  0.26 (0.05, 1.48) | .054  .129 |
| **Success** |  |  |  |  |  |  |
| Any | Structured vs unstructured | **.026** | URM status | URM  Non-URM | 4.72 (1.70,13.12)  1.12 (0.51,2.46) | **.003**  .779 |
| Any | Extended vs  Regular Dose | **.001** | Previous research submissions | Yes  No | 2.08 (1.07,4.04)  0.22 (0.05,1.06) | **.032**  .059 |
| Any R01 or R01-Like | Extended vs  Regular Dose | **.013** | Previous research submissions | Yes  No | 3.13 (1.22, 8.00)  0.16 (0.02, 1.38) | **.017**  .096 |

| **Panel C. Postdocs** |  |  |  |  |  |  |
| --- | --- | --- | --- | --- | --- | --- |
| **Submissions** |  |  |  |  |  |  |
| Any | Structured vs unstructured | **.009** | Previous training submissions | Yes  No | 0.25 (0.06,1.01)  3.24 (0.79,13.21) | .051  .102C |
| Any | Structured vs unstructured | **.012** | Previous research submissions | Yes  No | 0.16 (0.03,0.92)  2.37 (0.68,8.20) | **.041**  .174 |
| **Awards** |  |  |  |  |  |  |
| Any | Structured vs unstructured | **.046** | Previous research submissions | Yes  No | 0.26 (0.06,1.12)  3.08 (0.45,21.19) | .072  .253 |
| Any K | Structured vs unstructured | **.043** | Previous research submissions | Yes  No | 0.28 (0.06,1.34)  3.71 (0.53,26.21) | .111  .188 |
| **Success** |  |  |  |  |  |  |
| Any K | Structured vs unstructured | **.027** | Age | Q1 (age=29)  Q2 (age=33)  Q3 (age=37) | 0.20 (0.01,4.62)  3.99 (0.24,67.61)  80.39 (0.90, NE) | NA |
| Inter = interaction. URM = underrepresented in medicine. NA = not available from SAS LOGISTIC SLICE procedure; refer to confidence interval. NE = not estimable. SA = scientific advisor. | | | | | | |
