# Supplementary material for: Grant writing coaching groups: A randomized trial to discern the influences of coaching duration and engagement of scientific mentors on participants’ proposal submission and success rates
Source: PLoS One. 2026 Nov 10;21(11):e0355883. doi: 10.1371/journal.pone.0355883 (PMC13750578; doi:10.1371/journal.pone.0355883)
Supplement: Supplementary file 10 [file pone.0355883.s011.docx]

**Response to Reviewers, PONE-D-26-34663**

We reviewed the reference list to ensure it is complete and correct. None of the cited papers have been retracted. We added 1 new reference (#21), a manuscript currently under review by PLoS One (PONE-D-26045773), because it has become available as preprint on bioRxiv.

| Reviewer’s Comment | Response, incl description of revision (new text appears in green) | Location of revision in “clean” copy |
| --- | --- | --- |
| **Editor** | | |
| The reviewers were positive about the manuscript overall. One reviewer recommended acceptance without further revision, while the second found the manuscript informative, well written, and methodologically rigorous, particularly noting the value of the randomized study design. The latter reviewer raised two relatively minor points requiring clarification: additional details regarding institutional and participant recruitment, and an explanation of how potential contamination between study arms was prevented or minimized. I therefore recommend minor revision to address these points before acceptance. | We thank the academic editor for their comments. Below we address each point raised by the two reviewers. |  |
| Please provide an amended statement that declares *all* the funding or sources of support (whether external or internal to your organization) received during this study. (…) Please also include the statement “There was no additional external funding received for this study” in your updated Funding Statement. Please include your amended Funding Statement within your cover letter. We will change the online submission form on your behalf. | We added the following funding statement to the **cover letter:**  “This work was supported by the National Institutes of Health (NIH) National Institute of General Medical Sciences (NIGMS), grant number 7U01GM132366 (Principal Investigator Kolawole S Okuyemi). There was no additional external funding received for this study.”  Thank you for changing the online submission form on our behalf. | N/A |
| **Reviewer 1** | | |
| This work is a thorough report on the effectiveness of the method previously published by the authors. | We thank reviewer 1 for their thoughtful review and follow-up question. |  |
| I suggest that there is a definition of “broad similarities in research type” in the methods section, appears to be defined in Table 1. | We added the definition that is provided in Table 1 to the **Methods** section:  “Dyads were placed into coaching groups (4 to 5 dyads each) based on broad similarities in research type (ie, clinical, epidemiological/data science, laboratory based, social/behavioral research).” | Lines 151-152 |
| The number and type of institutions with participants would be helpful to the reader, e.g. participants were from xx R-1 and xx R2 institutions. | We added the following to the **Results** section:  “Ultimately, 367 individuals from 168 institutions were enrolled in the study and organized into 76 coaching groups.”  “A very high proportion of participants (>95%) were employed at free-standing medical schools, research institutes, or institutions with a Carnegie classification indicative of high or very high research activities and resources; only a very small number of faculty and no postdoctoral fellows were employed at Baccalaureate or Master’s Colleges and Universities. Nearly 10% (9.6%) of participants were employed at Minority-Serving Institutions. These and additional details about participants’ grant application and funding history were previously reported.” | 229  253-258 |
| I suggest addressing in limitations that the success rate is compared to national data, not the success rate of the institutions represented by the participants. | The **Limitations** section now reads:  “Study limitations include the lack of a true control group (ie, usual care: no coaching). It was the team’s judgement that having a usual care study arm was not ethical, given substantial data on the efficacy of the general coaching approach. Therefore, having 4 arms with treatments may have limited the ability to find between-arm differences, which is the rationale for using historical national NIH data to show overall improvement over usual care. However, we compared participants’ success rates to national NIH data as it was not possible to compare to the success rates at their home institutions. Because institutional success rates vary with research intensity and resources, the comparison with national data may overstate or understate the effect of the intervention relative to what participants would have achieved without it. Another limitation is that although arms were randomized, it is possible that arms were unbalanced at baseline on unmeasured variables, which, if adjusted as covariates, could have reduced residual variation and therefore either strengthened or weakened the between-arm differences. In addition, including faculty and postdocs in the same study limited the sample size and power for analyses of each separately.” | 601-605 |
| **Reviewer 2** | | |
| I appreciated the opportunity to review "Grant writing coaching groups: A randomized trial to discern the influences of coaching duration and engagement of scientific mentors on participants’ proposal submission and success rates." This is an informative manuscript that extends our knowledge about the effects of writing interventions for postdocs and early-career faculty. The manuscript expands on previous publications that laid the groundwork for the development of this enhanced group coaching intervention tested in this study. While the benefit of coaching and involvement of SAs seems somewhat intuitive, it is important to attempt to quantify these experimental factors, especially when the result is surprising. It is a deficit, in my opinion, that much of the research in the effectiveness of writing interventions does not meet standards for statistical rigor and is often just a description of a program with the finding that “we think it worked but we have no evidence except everyone liked it.” The effort to conduct a randomized trial, even if it could not compare a true control group, is important. I do not claim to be a statistician, although the tests and reporting seemed appropriate to me. This may be because the manuscript has the benefit of being very well written, making it easy for this reviewer to follow along and understand. I do have two questions that I would like the authors to address, in the hope that this will improve the manuscript. | We thank reviewer 2 for their generous comments and address their two questions below. |  |
| In the Methods section, beginning on line 124, the authors describe the study design, participant eligibility, and group randomization. I believe the manuscript would benefit from a description of study recruitment, especially given how successful recruitment was. How were the participating institutions chosen? Were they all CTSA institutions? Were they R1s? Were HBCUs specifically targeted? How were the study participants recruited? Did the authors target SAs and ask them to recruit students, or was a survey sent, for example? Please provide greater detail in this section. | The recruitment and randomization process is described in detail in our published protocol paper, referenced at the beginning of Methods. We added to the **Methods** section the following abbreviated description:  “We recruited a national US-based sample of early-career investigators (faculty and postdoctoral fellows) between October 1, 2019, and June 30, 2022, to participate in a pragmatic, unblinded, group-randomized trial (2x2 factorial design) to test variations of a group coaching intervention for the development of NIH and comparable national grant applications. Recruitment was conducted at the individual level through open national calls rather than through institutional partnerships; no institutions were selected or targeted. Study announcements were emailed to members of the NRMN community and to other individuals and biomedical professional organizations positioned to reach potential applicants, directing them to an application portal on an NRMN-sponsored webpage. Applicants completed a detailed online application survey and submitted their NIH biosketch, and members of the study team reviewed each application for eligibility. Eligible for inclusion were individuals without prior major independent research funding (eg, NIH R01) who were actively writing career development or research (K- or R-type) applications; had the necessary research background, preliminary data, and institutional resources to realistically compete for federal or comparable national funding; and had adequate time to commit to study participation. When cohorts were oversubscribed, men and individuals from racial and ethnic groups underrepresented in biomedical research were intentionally oversampled relative to the pool of eligible applicants.(13) We recruited 6 distinct cohorts, staggered to begin the trial in either January or June of 2020, 2021, and 2022.”  The **Results** section now also includes the statement regarding institutional type and number of institutions (see above). | 129-135  140-142 |
| How did you avoid contamination? Was there only one arm at any given site? Please provide some description. | We thank the reviewer for raising the issue of contamination. Because participants were recruited individually and coaching groups were composed by research area rather than by institution, some institutions were represented by more than 1 participant within the same cohort. However, we believe that the risk of contamination was low. Contamination is the study would occur if those in one arm were able to accidentally receive the benefit that those in another arm were receiving by design. The study design precluded that because all benefits were controlled by coaches, and coaches committed to the design in writing. If someone from the same institution in the unstructured arm talked to someone in the structured arm, even if the second person described the extra benefit of structured engagement of the scientific advisor, the person in the unstructured arm could not access the benefit because engagement depended on the coach: the coach would have constrained the scientific advisor of the participant in the unstructured arm from joining a group meeting or a 3-way conversation. Likewise, a participant in the regular dose arm could talk with someone in the extended dose arm and learn of the benefit of extended coaching, without receiving extended coaching from the coach, who would not provide additional coaching to participants in the regular arm.  We addressed the reviewer’s concert in the **Limitations** section:  “Finally, because coaching groups were composed by research area rather than institution, some participants of the same institution were enrolled in the same cohort and could have shared information across arms. However, the components that distinguished the arms (ie, extended coaching and structured SA engagement) could not be transferred through informal contact.” | 610-615 |
